# Investigating the effect of diet-induced prediabetes on the HPA-axis, sympathetic nervous system and endogenous opioids in male Sprague-Dawley rats

**DOI:** 10.3389/fendo.2026.1925577

**Published:** 2026-09-29

**Authors:** Nompumelelo Anna-Cletta Gumede, Zenele Chonco, Andile Khathi

**Affiliations:** Department of Human Physiology, School of Medicine, College of Health Sciences, University of KwaZulu-Natal, Durban, South Africa

**Keywords:** catecholamines, endogenous opioids, glucocorticoids, HPA-axis, metabolic stress, prediabetes, sympathetic nervous system

## Abstract

**Background:**

Prediabetes is associated with metabolic dysfunction that may disrupt neuroendocrine regulation before the onset of overt type 2 diabetes mellitus. Although previous studies have independently examined alterations in the hypothalamic–pituitary–adrenal (HPA) axis, sympathetic nervous system (SNS), and endogenous opioid system during prediabetes, the temporal interactions among these systems during disease progression remain poorly understood. This study aimed to investigate changes in these interconnected neuroendocrine pathways in a diet-induced prediabetic rats.

**Methods:**

Male Sprague-Dawley rats (n = 48) were exposed to a standard diet (non-prediabetic, n =24) or an HFHC diet (prediabetic, n =24) for 20 weeks. Prediabetes was confirmed using FBG, OGTT and HbA_1c_. Body weight, FBG, and OGTT were measured at weeks 20 to 32, prior to sacrifice. At each point, animals were sacrificed to assess plasma adrenocorticotropic hormone (ACTH), corticosterone, catecholamines (noradrenaline, adrenaline, and dopamine), and β-endorphin using ELISA. Hippocampal GR, MR, β_1_ AR, β_2_-AR, MOR, DOR, and KOR mRNA expression were quantified by qRT-PCR.

**Results:**

At week 20, the PD group exhibited marked reductions in circulating ACTH (30.70 ± 2.10 vs. 42.79 ± 2.91 pg/mL), corticosterone (16.89 ± 0.26 vs. 19.94 ± 0.24 pg/mL), noradrenaline (3.46 ± 0.15 vs. 4.36 ± 0.14 ng/mL), dopamine (1224.61 ± 24.83 vs. 1664.00 ± 19.43 pg/mL), and β-endorphin (376.72 ± 18.04 vs. 529.35 ± 9.70 ng/ml) compared with NPD group. These changes were accompanied by significant expressions of glucocorticoid and opioid receptors. By week 24, CORT, dopamine, and β-endorphin concentrations increased in the PD group compared with the NPD group, receptor transcripts were significantly downregulated, except for MOR, which was upregulated. At week 28, receptor expression remained downregulated. By week 32, plasma hormones and hippocampal receptor mRNA expression increased in the PD group. KOR was strongly inversely associated with β-endorphin in the NPD group at week 32.

**Conclusion:**

Diet-induced prediabetes is associated with progressive changes in hormones and hippocampal receptors of the HPA axis, SNS, and endogenous opioid system that become more pronounced with disease progression. Functional studies are required to determine whether these transcriptional and hormonal alterations translate into impaired neuroendocrine feedback regulation and are similar in other brain areas.

## Introduction

The hypothalamic-pituitary-adrenal axis (HPA-axis) and sympathetic nervous system (SNS) are interconnected neuroendocrine systems that maintain homeostasis (1). The HPA axis activity regulates the secretion of adrenocorticotropic hormone (ACTH) from the anterior pituitary and corticosterone (CORT) from the adrenal cortex, while the SNS releases catecholamines from the adrenal medulla and the sympathetic nerve terminals (2, 3). Corticosterone inhibits pancreatic insulin secretion, decreases glucose uptake, while promoting hepatic gluconeogenesis, thus increasing blood glucose (4, 5). Circulating CORT exerts regulatory feedback within the brain by binding to high-affinity mineralocorticoid receptors (MR) and lower-affinity glucocorticoid receptors (GR) (6). Catecholamines inhibit insulin-mediated glucose uptake by inhibiting insulin signaling, thereby impairing the translocation of glucose transporter (GLUT)-4 to the plasma cell membrane (7). Central and peripheral catecholamines exert their physiological effects via β_1_- and β_-_ adrenergic receptors (AR), which modulate neurovascular and metabolic functions (8). The endogenous opioid system acts through β-endorphin to attenuate the HPA-axis and SNS overactivation (9, 10). It achieves this by binding mu (µ; MOR), kappa (κ; KOR) and delta (δ; DOR) opioid receptors widely expressed in the brain (11, 12). Although these neuroendocrine systems are essential for maintaining homeostasis during stress, prolonged dysregulation of stress-responsive pathways has been associated with metabolic disturbances and the development of type 2 diabetes mellitus (T2DM) (13). T2DM is a metabolic disorder caused by defective insulin secretion from the pancreas and the inability of insulin-sensitive tissues to respond adequately to insulin (14). In T2DM, basal cortisol is elevated and sustained elevations have been associated with hyperglycemia and increased glycated hemoglobin (HbA_1c_) levels (15). Furthermore, impaired stress responsivity in T2DM is associated with altered MR and GR sensitivity (16). The expression of the MOR is downregulated in T2DM (17). Although endogenous opioids like β-endorphin have been shown to facilitate glucose uptake in T2DM, the status and temporal changes of these interconnected neuroendocrine systems prior to the development of overt diabetes remain poorly understood (18, 19).

The onset of T2DM is often preceded by prediabetes, a state in which blood glucose levels are above normal but below the diagnosis threshold for T2DM (20). Using a high-fat high-carbohydrate (HFHC) diet-induced rodent model of prediabetes, a previous study demonstrated HPA-axis dysregulation and impaired stress coping (21). Furthermore, autonomic neuropathy and cardiovascular autonomic neuropathy, which are associated with SNS dysfunction, are prevalent in individuals with impaired fasting glucose (IFG) (22). Although HPA-axis and SNS alterations have been described during prediabetes, the endogenous opioid system has received comparatively less attention. Moreover, these systems have largely been investigated independently, and few studies have examined their temporal relationship as prediabetes progresses. It therefore remains unclear whether progression of prediabetes is accompanied by coordinated changes in HPA-axis activity, sympathetic activity, and endogenous opioid signaling. Therefore, the present study investigated temporal changes in circulating markers of the HPA axis, SNS activity, and the endogenous opioid system, along with their corresponding receptor expression, in male Sprague-Dawley rats with HFHC diet-induced prediabetes.

## Materials and methods

### Animals

Sample size was determined *a priori* using G*Power version 3.1.9.2. For the 2 × 4 factorial design comprising two dietary groups and four time points, an effect size of *f* = 0.55, an α level of 0.05, and a statistical power of 0.80 were specified for the Group × Time interaction. The effect size was selected *a priori* to represent a relatively large anticipated interaction effect and was not derived from pilot data. The calculation yielded a minimum required sample size of approximately 41 animals. To ensure equal allocation across the eight experimental cells, the sample size was increased to 48 animals, corresponding to six animals per cell. Therefore, 48 male Sprague-Dawley (150-180g) rats were housed at the Biomedical Research Unit (BRU) of the University of KwaZulu-Natal (UKZN). The animals were kept under standard experimental conditions at room temperature (22 ± 2 °C), humidity (55 ± 5%), and 12-hour day: 12-hour night cycle. The animals consumed a standard rat chow consisting of carbohydrates (35% kcal/g), fats (15%kcal/g) and proteins (30% kcal/g) (Meadow Feeds, South Africa) and water *ad libitum* for two weeks to acclimatize before being exposed to an experimental diet, the high-fat, high-carbohydrate diet (HFHC). The high-fat-high-carbohydrate (HFHC) diet was composed of carbohydrates (55% kcal/g), fats (30% kcal/g), proteins (15% kcal/g) and water supplemented with 15% fructose. All experimental procedures were conducted in accordance with the ethics and animal care guidelines of the UKZN Animal Research Ethics Committee (AREC), Durban, South Africa. (Ethics No: *AREC/00005695/2023*).

### Experimental design

After two weeks of acclimatization, the animals were grouped into Group A (n = 24) and Group B (n = 24) and housed in pairs. Group A consumed a standard rat chow and water *ad libitum.* Group B consumed an HFHC diet and water supplemented with fructose (15%) for 20 weeks. After 20 weeks, The fasting blood glucose (FBG) concentrations of 5.6–6.9 mmol/L, oral glucose tolerance test (OGTT) 2-h postprandial glucose concentration of 7.8–11.0 mmol/L, and glycated hemoglobin (HbA_1c_) concentration were used to diagnose prediabetes. These parameters are aligned with the ADA clinical criteria for human diagnosis of prediabetes. Blood glucose was measured by the tail-prick method using a One-Touch Select glucometer (Lifescan, Malta, United Kingdom). OGTT was performed according to a previously established protocol (23). Briefly, after a 12-hour fasting period, fasting blood glucose (FBG) was measured at 08:30 in the morning, this marked time 0 minutes in all the animals. At 09:00, glucose (0.86g/kg, p.o.) was loaded into the animals via oral gavage (18-gauge gavage needle, 38 mm long, curved with 21/4mm ball end). Glucose concentrations were measured at 15-, 30-, 60- and 120-min following glucose loading. HbA_1c_ concentration was measured using a commercially available ELISA kit. The assay reports HbA1c as an absolute concentration in ng/mL rather than as a percentage of total hemoglobin. As total hemoglobin was not measured, the HbA_1c_ concentrations obtained from the ELISA could not be converted to percentage HbA_1c_. Accordingly, the human ADA HbA_1c_ threshold of 5.7–6.4% was not applied to the ELISA-derived HbA1c measurements. Animals in Group A were found to be non-prediabetic and termed NPD, while the animals in Group B were found to be prediabetic and termed PD.

### Blood collection and tissue harvesting

To establish baseline changes that occur in the HPA-axis, SNS, and endogenous opioid system at prediabetes induction (week 20), six animals from each experimental group were humanely euthanized by decapitation at 08:30 in the morning using a sharp guillotine. To assess the changes that occur as prediabetes progresses, an additional six animals from each group were euthanized after four weeks, that is, at weeks 24, 28, and 32, using the same procedure. Immediately after euthanasia, trunk blood was collected and the brain was rapidly excised. The tissues were rinsed in cold saline, snap-frozen in liquid nitrogen, and stored at -80 °C in a Biofreezer (Snijders Scientific, Tilburg, The Netherlands) until further analysis. Whole blood was also collected, with a portion stored at -4 °C for HbA1c measurement. The remaining blood was centrifuged (Eppendorf centrifuge 5403, Germany) at 503 x *g* for 15 min at 4 °C to separate plasma. Plasma was aliquoted and stored at − 80 °C in a Biofreezer until biochemical analyses were performed. Plasma samples were subsequently used for the quantification of ACTH, corticosterone, norepinephrine, epinephrine, dopamine, and β-endorphin by enzyme-linked immunosorbent assay (ELISA), whereas hippocampal tissue was used for RNA extraction, cDNA synthesis, and quantitative real-time polymerase chain reaction (RT-qPCR) analysis of glucocorticoid receptor (GR), mineralocorticoid receptor (MR), β1-adrenergic receptor (β1-AR), β2-adrenergic receptor (β2-AR), μ-opioid receptor (MOR), δ-opioid receptor (DOR), and κ-opioid receptor (KOR) gene expression.

### Biochemical analysis

Glycated hemoglobin (HbA_1c_; RK03710) was measured by sandwich ELISA according to the manufacturer’s protocol (ABclonal Technology Co., Ltd., Woburn, MA, USA). Briefly, 100 µL of the standards and samples were loaded in duplicate into pre-coated micro-ELISA plate wells and incubated at 37 °C for 90 minutes. Following removal of the liquid, 100 µL of biotinylated detection antibody working solution was added to each well, and the plate was incubated at 37 °C for 60 min. The wells were then washed three times with wash buffer before adding 100 µL of horseradish peroxidase (HRP)-conjugate working solution. After incubation at 37 °C for 30 min, the wells were washed five times, and 90 µL of substrate solution was added. The plate was incubated at 37 °C for 15 min in the dark, after which the reaction was terminated by adding the stop solution, resulting in a yellow color. Optical density was measured at 450 nm using a Spectrostar Nano microplate reader (BMG LABTECH, Ortenberg, Germany). The average absorbance of duplicate wells was calculated by subtracting the blank value, and analyte concentrations were determined by interpolation from the corresponding standard curves.

Adrenocorticotropic hormone (ACTH: E-EL-R0048), corticosterone (CORT: E-EL-0160), epinephrine (EPI: E-EL-0045), norepinephrine (NE: E-EL-0047), dopamine (E-EL-0046) and β-endorphin (E-EL-R0105) were measured in plasma using their respective competitive ELISA kits according to the manufacturer’s instructions (Elabscience Biotechnology Co., Ltd., Houston, TX, USA). Briefly, 50 µL of standard, blank, or sample was added to microplate wells pre-coated with rat ANG-II, followed by the addition of 50 µL of biotinylated detection antibody working solution. The plate was sealed and incubated at 37 °C for 45 min. Following incubation, the reaction mixture was discarded, and the wells were washed three times with wash buffer. Subsequently, 100 µL of horseradish peroxidase (HRP)-conjugate working solution was added to each well, and the plate was sealed and incubated at 37 °C for 30 min. The wells were then washed five times before adding 90 µL of substrate solution and incubating at 37 °C for 15 min in the dark. The reaction was terminated by adding the stop solution, resulting in a yellow color. Optical density was measured at 450 nm using a Spectrostar Nano microplate reader (BMG LABTECH, Ortenberg, Germany). The average absorbance of duplicate wells was calculated after subtraction of the blank value, and concentrations were determined by interpolation from the corresponding standard curve.

### RNA extraction

Ribonucleic acid (RNA) was extracted from the hippocampus (10mg) using the Maxwell^®^ RSC simply RNA Tissue kit according to the manufacturer’s protocol (Promega Corporation, Madison, WI, USA). Briefly, hippocampal tissue was homogenized in the lysis buffer provided with the kit in the presence of 1-thioglycerol to inactivate RNases. The lysate was processed with Proteinase K to facilitate tissue digestion before being transferred to the Maxwell^®^ RSC cartridges. Automated RNA purification was performed using the Maxwell^®^ RSC Instrument, which employs paramagnetic particle technology to isolate total RNA. Following a series of automated washing steps, purified RNA was eluted in 50 µl of nuclease-free water. The concentration and purity of the isolated RNA were determined spectrophotometrically using a NanoDrop 1000 spectrophotometer (Thermo Fisher Scientific Inc, Massachusetts, USA) by measuring absorbance at 260 and 280 nm. Purified RNA samples were stored at −80 °C until further analysis.

### cDNA synthesis

Complementary DNA (cDNA) was synthesized from 2 µg of total RNA using the Transcriptor Universal cDNA Master (Roche Diagnostics GmbH, Mannheim, Germany) according to the manufacturer’s instructions. Briefly, total RNA (5µl) was combined with the Transcriptor Universal Reaction Buffer (4µ), Transcriptor Universal Reverse Transcriptase (1µl), and PCR-grade water to make a 20 µl final volume. Reverse transcription was performed in a thermal cycler under the manufacturer’s recommended conditions to generate first-strand cDNA. The synthesized cDNA was stored at −20 °C until use for quantitative real-time polymerase chain reaction (RT-qPCR) analysis.

### Quantitative real-time polymerase chain reaction (RT-qPCR)

Gene expression analysis was performed by quantitative real-time polymerase chain reaction (RT-qPCR) using the Applied Biosystems SYBR™ Green Universal Master Mix, as per the protocol (Thermo Fisher Scientific Inc., Massachusetts, USA). Amplification reactions were prepared by combining SYBR™ Green Universal Master Mix, gene-specific forward and reverse primers, cDNA template, and nuclease-free water to the required reaction volume (50µl). Real-time PCR amplification was performed using the LightCycler^®^ 96 Instrument (Roche Diagnostics GmbH, Mannheim, Germany) under the recommended cycling conditions, consisting of an initial polymerase activation step followed by 40 cycles of denaturation, annealing, and extension. A melt curve analysis was performed at the end of each run to verify amplification specificity. GAPDH was used as the housekeeping gene, TGGATTTGGACGCATTGGTC (forward) and TTTGCACTGTACGTGTTGAT (reverse). The sequences of the oligonucleotides that were used are shown in **Table 1**. Relative gene expression was calculated using the comparative 2^-ΔΔCt^ method, with the target genes normalized to the housekeeping gene.

**Table 1:** Oligonucleotide sequence of the receptors.

Sequence name	Sequence
Nr3c1 gene (glucocorticoid receptor)	Forward: 5′-ACC TCG ATG ACC AAA TGA CC-3′ Reverse: 5′-AGC AAA GCA GAG CAG GTT TC-3′
NR3c2 gene (mineralocorticoid receptor)	Forward: 5′-AAA GGG TAG TGT GTG CAG GG-3′ Reverse: 5′-GTT CTC CTA GTT CCC GGA GG-3′
β_1_-AR	Forward: 5’-AACGAGCGGATCATGCTTTC-3’, Reverse: 5’- AATGCATATGACATGCACACGAT-3’
β_2_-AR	Forward: 5′GAGCACAAAGCCCTCAAGAC3′ Reverse: 5′GTTGACGTAGCCCAACCAGT3′
mu	Forward: 5’-ATCGGGCTCCAAAGAAAAGG-3’, Reverse: 5’-CATCCAGGAAGGCGTAAAGAAC-3’
delta	Forward primer: 5’-GCTGGGCTACGCCAACAG-3’, Reverse primer: 5’-CGGAAGCAGCGCTTGAAG-3’
kappa	Forward: 5’- TCTTTATCCTGGTCGAGGCTCTA-3’ Reverse: 5’- CCCAAGGCAATGCAGAAGTAA-3’

### Statistical analysis

All data were expressed as means ± S.E.M. Statistical comparisons were performed with GraphPad InStat Software (version 8.00, GraphPad Software, Inc., San Diego, California, USA). Normality was tested using the Shapiro–Wilk test. The differences in body weight, fasting blood glucose and oral glucose tolerance were measured before sacrifice at week 20, 24, 28 and 32. As different animals were sacrificed at each time point, these measurements were obtained from independent animals, with progressively decreasing sample sizes across the experimental period. Therefore, differences between the NPD and PD groups at each time point were assessed using the unpaired Student’s *t*-test. The differences in terminal blood and hippocampal tissue measurements of glycated hemoglobin, catecholamines, glucocorticoids, and endogenous opioids were analyzed using an ordinary two-way ANOVA, with experimental group (NPD vs PD) and time (weeks 20, 24, 28, and 32) as the two factors. The analysis assessed the main effects of group and time, as well as the group × time interaction. For significant effects, Sidak’s multiple-comparisons test was used to perform pairwise comparisons between the NPD and PD groups at each time point, with correction applied across the four time-specific comparisons for each outcome. Pearson’s correlation analysis was performed for normally distributed variables, whereas Spearman’s rank correlation was used for variables that did not meet assumptions of normality. Correlation analysis was performed separately within the NPD and PD groups at each time point to assess relationships between circulating biomarkers and corresponding hippocampal receptor mRNA expression, including CORT with GR and MR; NE and EPI with β1-AR and β2-AR; and β-endorphin with MOR, KOR, and DOR. Correlation coefficients (r for Pearson’s and ρ for Spearman’s correlation) were used to describe the strength and direction of the associations. To account for multiple testing in the correlation analyses, the false discovery rate (FDR) was controlled at 5% using the two-stage step-up procedure of Benjamini, Krieger and Yekutieli, as implemented in GraphPad Prism. A p-value of *p* < 0.05 was considered statistically significant.

## Results

### Body weight

Body weight was measured in weeks 20, 24, 28, and 32 in the NPD and PD groups. **Table 2** shows that body weight was significantly higher in the PD group than in the NPD group from week 20 to 32.

**Table 2:** Body weight (g) between the NPD and PD groups measured at week 20, 24, 28 and 32.

Weeks	NPD	PD	*p*
20 (n= 24 per group)	483.10 ± 11.05	525.50 ± 17.77*	0.0497
24 (n = 18 per group)	479.40 ± 9.57	536.70 ± 12.70*	0.0010
28 (n = 12 per group)	484.60 ± 9.28	553.30 ± 8.51*	<0.0001
32 (n = 6 per group)	470.30 ± 5.08	589.80 ± 9.32*	<0.0001

*Indicates statistical significance. NPD, non-prediabetes; PD, prediabetes. Values are presented as mean ± SEM.

### Fasting blood glucose (FBG)

Fasting blood glucose (FBG) was measured in weeks 20, 24, 28 and 32 in the NPD and PD groups. **Table 3** shows that the FBG was significantly higher in the PD group than in the NPD group during weeks 20-32. The average FBG in the NPD falls below the prediabetes diagnosis range (5.6 – 6.9 mmol/L), whereas it falls within the range in the PD. However, at week 28, FBG in the PD group was below the diagnosis range though still higher than in the NPD group.

**Table 3:** Fasting blood glucose (mmol/L) between the NPD and PD groups at week 20, 24, 28 and 32.

Weeks	NPD	PD	*p*
20 (n = 24 per group)	5.13 ± 0.11	5.99 ± 0.20*	0.0074
24 (n = 18 per group)	5.21 ± 0.10	6.23 ± 0.10 *	<0.0001
28 (n = 12 per group)	4.55 ± 0.07	5.4 ± 0.03*	<0.0001
32 (n = 6 per group)	5.00 ± 0.07	5.97 ± 0.17*	0.0004

*Indicates statistical significance. NPD, non-prediabetes, PD, prediabetes. Values are presented as mean ± SEM.

### Oral glucose tolerance test (OGTT)

An oral glucose tolerance test was conducted at weeks 20, 24, 28, and 32 between the NPD and PD groups. **Figure 1** shows results obtain at (A) week 20, (B) week 24, (C) week 28 and (D) week 32. Blood glucose levels in the PD group remained higher than in the NPD group from week 20 through week 32.

**Figure 1:**
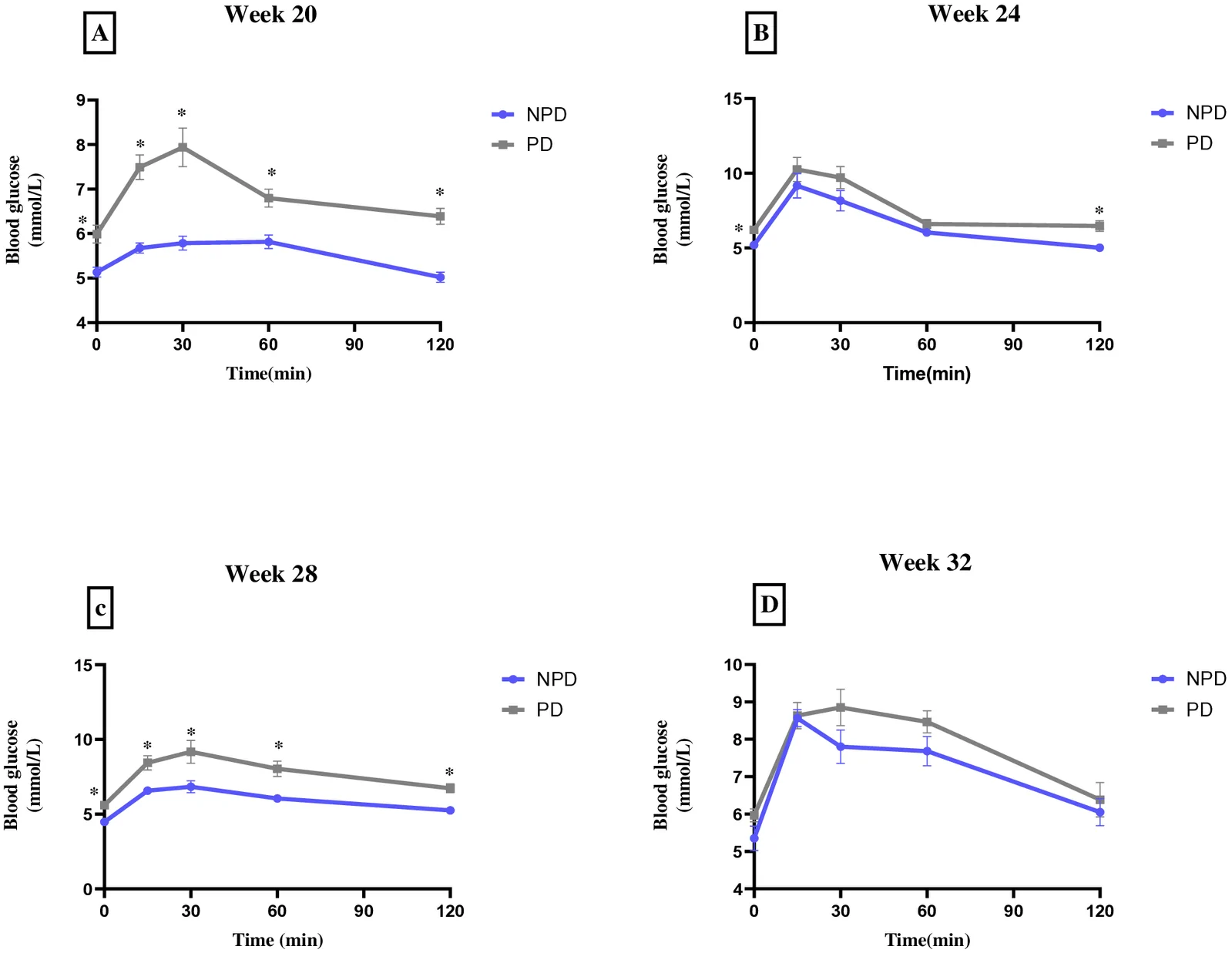
Oral glucose tolerance test between the NPD and PD groups at **(A)** week 20, **(B)** week 24, **(C)** week 28 and **(D)** week 32. Values are presented as mean ±SEM. *Indicates statistical significance. NPD, nonprediabetes; PD, prediabetes.

### Glycated hemoglobin (HbA_1c_)

A two-way ANOVA was performed to evaluate the effects of diet group (NPD vs PD) and time (weeks 20-32) on HbA1c concentration. There was no significant group × time interaction (*F* (3,40) = 1.08, *p* = 0.3686, η² = 0.075), indicating that changes in HbA1c over time did not differ between groups. There was also no significant main effect of time (*F* (3,40) =0.1574, *p* = 0.9243, η²=0.012). However, a significant main effect of group was observed (*F* (1,40) =78.60, *p* < 0.0001, η²=0.663), with the PD group (206.5 ng/mL) exhibiting higher HbA1c concentrations than the NPD group (197.6 ng/mL) overall (mean difference = 8.81, 95% CI 6.80-10.82). In **Figure 2**, Sidak’s *post hoc* comparisons revealed that the PD group had significantly higher HbA1c concentration than the NPD group at week 20 (*p* = 0.0248), week 24 (*p* < 0.0001), week 28 (*p* < 0.0001), and week 32 (*p* < 0.0001).

**Figure 2:**
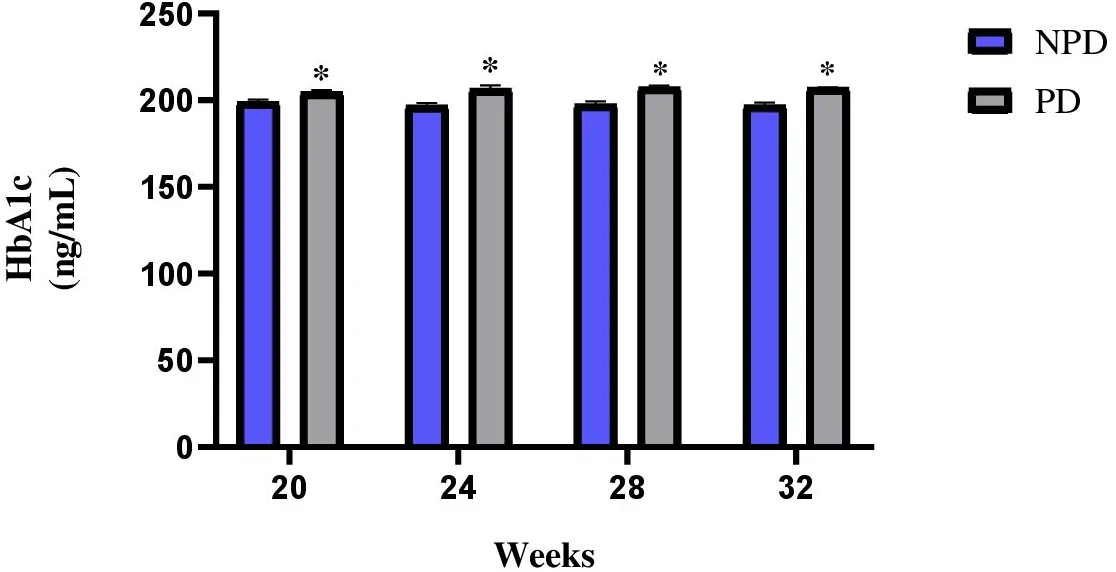
Concentration of HbA1c in the NPD and PD groups (n=6 per group). Values are presented as mean ± SEM. *Indicates statistical significance. NPD, non-prediabetes; PD, prediabetes; HbA1c, glycated hemoglobin.

### Glucocorticoids

#### Adrenocorticotropin hormone (ACTH)

A two-way ANOVA was conducted to determine the effects of diet group (NPD vs PD) and time (weeks 20-32) on ACTH concentrations. There was a significant interaction between groups and time (F(3,40) = 6.704, *p* = 0.0009, η² = 0.335), indicating that changes in ACTH concentrations over time differed between groups. A significant main effect of time was also observed (F(3,40) = 7.544, *p* = 0.0004, η² = 0.361), indicating that ACTH concentrations varied across the study period. However, the overall effect of the group was not significant (F(1,40) = 0.8275, *p* = 0.3685, η² = 0.020). In **Figure 3**, Sidak’s *post hoc* comparisons revealed that ACTH concentrations were significantly lower in the PD group (30.70 ± 2.10 pg/mL) than in the NPD group (42.79 ± 2.91 pg/mL) at week 20 (*p* = 0.0008), whereas no significant differences were detected at weeks 24, 28, or 32 (p>0.05).

**Figure 3:**
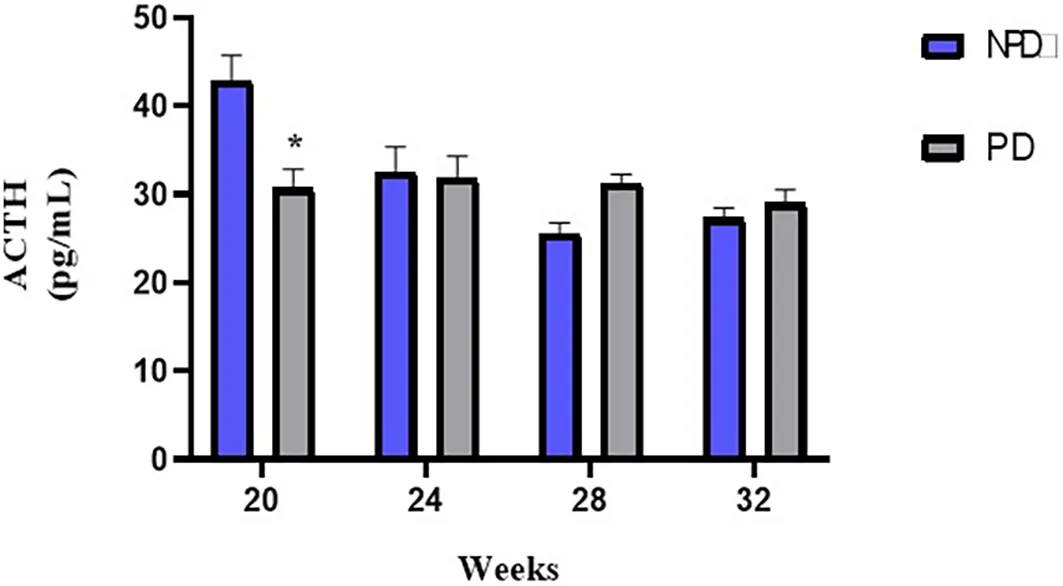
Concentration of adrenocorticotropin hormone in the NPD and PD groups (n=6 per group). The values are expressed as mean ± SEM. *Indicates statistical significance. NPD, non-prediabetes; PD, prediabetes; ACTH, adrenocorticotropin hormone.

#### Corticosterone (CORT)

A two-way ANOVA was performed to assess the effects of dietary group (NPD vs PD) and time (weeks 20-32) on CORT concentrations. There was a highly significant interaction between group and time (*F*(3,40) = 39.00, *p* < 0.0001, η² = 0.745), indicating that changes in CORT concentrations over time differed between groups. A significant main effect of time was also observed (*F* (3,40) =27.54, *p* < 0.0001, η²=0.674), demonstrating temporal changes in CORT concentrations throughout the study. However, there was no significant overall effect of group (*F* (1,40) = 0.1406, *p* = 0.7097, η²=0.003). In **Figure 4**, Sidak’s *post hoc* comparisons revealed significant differences between NPD and PD groups at week 20 (NPD: 19.94 ± 0.24 ng/mL and PD: 16.89 ± 0.26 ng/mL, *p* < 0.0001), week 24 (NPD: 17.54 ± 0.01 ng/mL and PD: 18.86 ± 0.26 ng/mL, *p* = 0.0013), and week 32 (NPD:16.29 ± 0.05 ng/mL and PD:17.52 ± 0.19 ng/mL, *p* = 0.0028), whereas no significant difference was detected at week 28 (NPD: 16.32 ± 0.32 ng/mL and PD:17.07 ± 0.34 ng/mL, *p* = 0.1135).

**Figure 4:**
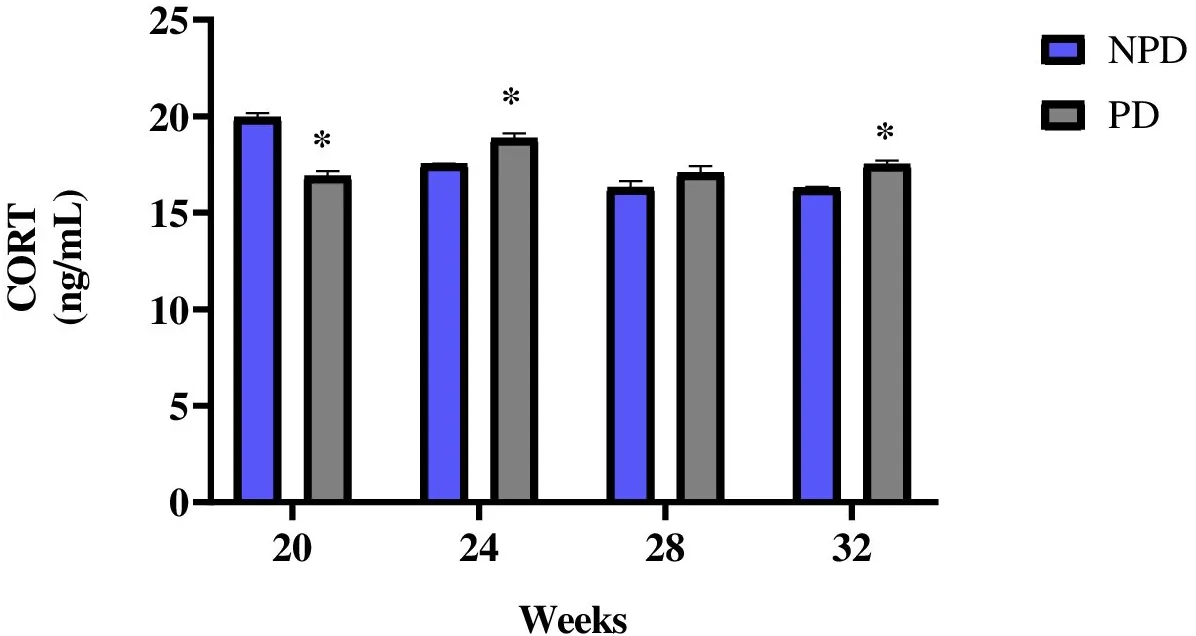
Concentration of corticosterone in the NPD and PD groups (n = 6 per group). The values are expressed as mean ± SEM. *Indicates statistical significance. NPD, non-prediabetes; PD, prediabetes; CORT, corticosterone.

#### Correlation between plasma adrenocorticotropin hormone (ACTH) and corticosterone (CORT) concentration.

Correlation analysis in **Table 4** shows that in the NPD group, ACTH and COR concentrations were strongly and positively correlated (r = 0.92, 95% CI: 0.41-0.99, *p* = 0.0101) at week 20; however, this association did not remain significant following FDR correction (*q* = 0.0848). In the PD group, ACTH and CORT concentrations showed a non-significant negative correlation across all weeks.

**Table 4:** Correlation between plasma ACTH and CORT concentrations in the NPD and PD group at weeks 20, 24, 28 and 32.

Week	Group (n = 6 per group)	Correlation coefficient	95% CI	*p*-value	*q-*value
20	NPD	r = 0.92	0.41- 0.99	0.0101	0.0848
	PD	r = -0.60	-0.95 - 0.41	0.2080	0.5255
24	NPD	r = -0.67	-0.96 - 0.32	0.1485	0.5225
	PD	r = -0.30	-0.89 - 0.68	0.5612	0.5893
28	NPD	r = -0.49	-0.93 – 0.54	0.3263	0.5225
	PD	r = -0.34	-0.90 – 0.65	0.5041	0.5893
32	NPD	r = 0.45	-0.57 – 0.92	0.3732	0.5225
	PD	r = -0.55	-0.94 – 0.48	0.2610	0.5225

Data are presented as correlation coefficient, 95% CI, *p*-value and FDR-adjusted *q*-value (n = 6 per group).

#### Mineralocorticoid (MR) and Glucocorticoid (GR) Receptor

A two-way ANOVA was performed to evaluate the effects of dietary group (NPD vs PD) and time (weeks 20-32) on GR and MR mRNA expression. In **Figure 5A**, there was a significant interaction between group and time (F (3,40) =50.56, *p* < 0.0001, η²=0.791), indicating that temporal changes in GR expression differed between groups. Significant main effects of time (F (3,40) =48.55, *p* < 0.0001, η²=0.785) and group (F (1,40) =31.25, *p* < 0.0001, η²=0.439) were also observed. Overall, GR mRNA expressions were significantly higher in the PD group than in the NPD group (PD: 1.918 and NPD: 1.157; mean difference = 0.761, 95% CI 0.486-1.035). Sidak’s *post hoc* comparisons revealed that GR mRNA expression was significantly higher in the PD group than in the NPD group at week 20 (*p* < 0.0001) and week 32 (*p* < 0.0001) and significantly lower at week 28 (*p* = 0.0066). No significant difference was observed in week 24 (*p* = 0.0993). In **Figure 5B**, a significant interaction between groups and time was observed (F (3,40) = 19.72, p < 0.0001, η²=0.597), indicating that temporal changes in MR mRNA expression differed between groups. There was also a significant main effect of time (F(3,40) = 91.29, *p* < 0.0001, η²=0.873), whereas the overall effect of group was not significant (F(1,40) = 2.398, *p* = 0.1293, η²=0.057). Sidak’s *post hoc* analysis revealed no significant difference between groups at week 20 (*p* = 0.8865). However, MR expression was significantly lower in the PD group than in the NPD group at week 24 (*p* = 0.0126) and week 28 (*p* = 0.0002). By week 32, the PD group exhibited significantly higher MR expression than the NPD group (*p* < 0.0001).

**Figure 5:**
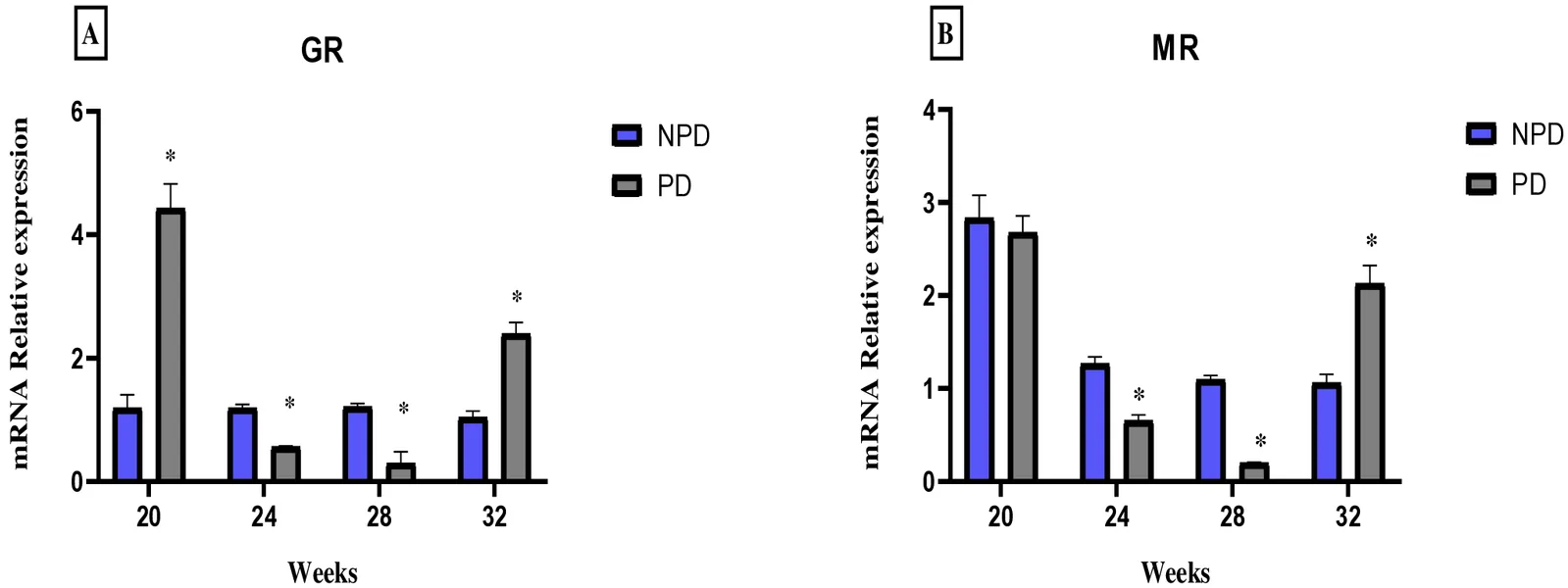
The expression of **(A)** glucocorticoid and **(B)** mineralocorticoid receptors in the NPD and PD groups (n = 6 per group). Values are expressed as mean ± SEM. *Indicates statistical significance. NPD, non-prediabetes; PD, prediabetes; GR, glucocorticoid receptor; MR, mineralocorticoid receptor.

#### Correlation between plasma corticosterone (CORT) concentration, hippocampal glucocorticoid (GR) and mineralocorticoid (MR) mRNA expression.

Correlation analysis in **Table 5** revealed no statistically significant correlations between CORT and GR expression at any time point in either group. CORT was positively correlated with MR expression in the NPD group at week 24 (r = 0.89, 95% CI: 0.29-0.99, *p* = 0.0168), whereas CORT was strongly negatively correlated with MR expression in the PD group at week 32 (r = −0.94, 95% CI: -0.99- -0.52, *p* = 0.0059). These associations did not remain statistically significant following FDR correction (*q* = 0.1411 and *q* = 0.0991, respectively).

**Table 5:** Correlation between plasma corticosterone (CORT) concentration and hippocampal glucocorticoid receptor (GR) and mineralocorticoid receptor (MR) mRNA expression in non-prediabetic (NPD) and prediabetic (PD) rats at weeks 20, 24, 28 and 32.

Week	Group (n = 6 per group)	Correlation coefficient	95% CI	*p*-value	*q-*value
GR					
20	NPD	r = 0.46	-0.56-0.93	0.3616	0.7639
	PD	r = 0.41	-0.60-0.92	0.4201	0.7639
24	NPD	r = 0.14	-0.75-0.86	0.7868	0.9442
	PD	r = 0.41	-0.60-0.92	0.4246	0.7639
28	NPD	r = -0.15	-0.86-0.75	0.7797	0.9442
	PD	r = 0.35	-0.65-0.90	0.5002	0.7649
32	NPD	r = 0.08	-0.78-0.84	0.8755	0.9806
	PD	r = -0.78	-0.98-0.07	0.0644	0.3606
MR					
20	NPD	r = 0.35	-0.65-0.90	0.4990	0.7639
	PD	r = 0.47	-0.55-0.93	0.3447	0.7639
24	NPD	r = 0.89	0.29-0.99	0.0168	0.1411
	PD	r = 0.68	-0.30-0.96	0.1402	0.5060
28	NPD	r = -0.66	-0.96-0.32	0.1506	0.5060
	PD	r = 0.03	-0.80-0.82	0.9603	1.0000
32	NPD	r = -0.20	-0.87-0.73	0.7032	0.9442
	PD	r = -0.94	-0.99- -0.52	0.0059	0.0991

Data are presented as correlation coefficient, 95% CI, *p*-value and FDR-adjusted *q*-value (n = 6 per group).

### Catecholamines

#### Norepinephrine (NE)

A two-way ANOVA was conducted to evaluate the effects of dietary group (NPD vs PD) and time (weeks 20-32) on NE concentrations. A significant interaction between group and time was observed (F(3,40) = 8.159, *p* = 0.0002, η²=0.380), indicating that temporal changes in NE concentrations differed between groups. Significant main effects of time (F (3,40) =16.19, *p* < 0.0001, η²=0.548) and group (F (1,40) =8.689, *p* = 0.0053, η²=0.178) were also detected. Overall, NE concentrations were significantly higher in the NPD group than in the PD group (NPD: 4.296 and PD: 3.896; mean difference = 0.400, 95% CI: 0.126-0.674). In **Figure 6**, Sidak’s *post hoc* comparisons showed significantly higher NE concentrations in the NPD group at week 20 (NPD:4.36 ± 0.14 ng/mL and PD: 3.46 ± 0.15 ng/mL, *p* = 0.0085) and week 28 (NPD: 4.69 ± 0.27 ng/mL and PD: 3.81 ± 0.21 ng/mL, *p* = 0.0093), whereas no significant difference was observed at week 24 (NPD: 5.04 ± 0.22 ng/mL and PD: 4.48 ± 0.18 ng/mL, *p* = 0.1622). At week 32, NE concentrations were significantly (*p* = 0.0371) higher in the PD group (3.84 ± 0.18 ng/mL) than in the NPD group (3.10 ± 0.15 ng/mL).

**Figure 6:**
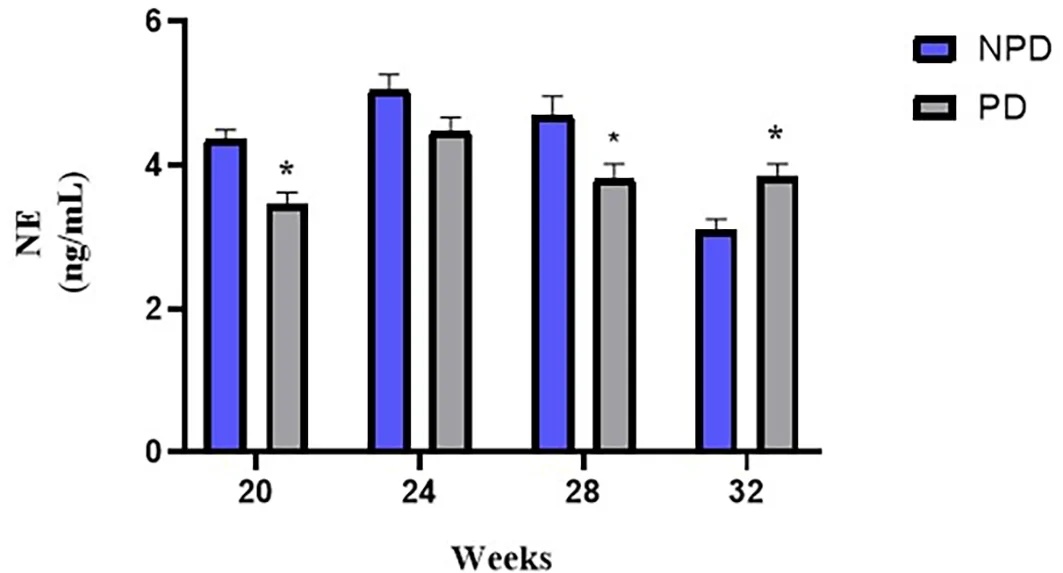
Norepinephrine between the NPD and PD groups (n = 6 per group). Values are expressed as mean ± SEM. *Indicates statistical significance. NPD, non-prediabetes; PD, prediabetes; NE, norepinephrine.

#### Epinephrine (EPI)

A two-way ANOVA was performed to investigate the effects of dietary group (NPD vs PD) and time (weeks 20-32) on EPI concentrations. A significant interaction between group and time was observed (F (3,40) =35.18, *p* < 0.0001, η²=0.725), indicating that changes in EPI concentrations over time differed between groups. A significant main effect of time was also detected (F (3,40) =9.042, *p* = 0.0001, η²=0.404), whereas the overall effect of group was not significant (F (1,40) =0.007, *p* = 0.9337, η²<0.001). In **Figure 7**, Sidak’s *post hoc* comparisons revealed no significant differences between NPD and PD groups at weeks 20 (*p* = 0.1831) and 24 (*p* = 0.6737). However, EPI concentrations were significantly (*p* < 0.0001) higher in the NPD group (856.68 ± 14.21 pg/mL) than in the PD group (599.60 ± 15.52 pg/mL) at week 28 and significantly (*p* < 0.0001) higher in the PD group (816.87 ± 20.80 pg/mL) than in the NPD group (661.26 ± 27.72 pg/mL) at week 32.

**Figure 7:**
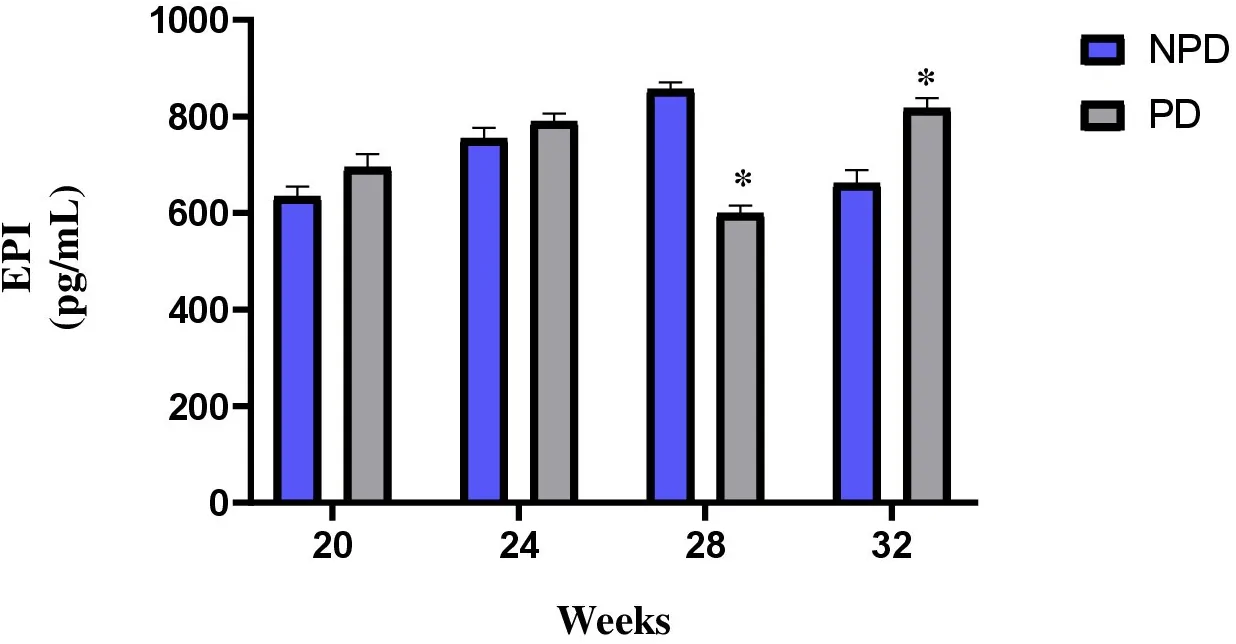
Epinephrine concentration in the NPD and PD group (n 6 per group). Values are expressed as mean ± SEM. *Indicates statistical significance. NPD, non-prediabetes; PD, prediabetes; EPI, epinephrine.

#### Dopamine

A two-way ANOVA was performed to evaluate the effects of dietary group (NPD vs PD) and time (weeks 20-32) on dopamine concentrations. There was a significant interaction between groups and time (F (3,40) =45.23, *p* < 0.0001, η²=0.772), indicating that changes in dopamine concentrations over time differed between groups. A significant main effect of time was also observed (F (3,40) =9.058, *p* = 0.0001, η²=0.405), whereas the overall effect of group was not significant (F (1,40) =0.2267, *p* = 0.6366, η²=0.006). In **Figure 8**, Sidak’s *post hoc* analysis showed that dopamine concentrations were significantly (p<0.0001) higher in the NPD group (1664 ± 19.43 pg/mL) than in the PD group (1224.61 ± 24.83 pg/mL) at week 20. However, dopamine concentrations were significantly higher in the PD group at weeks 24 (NPD: 1132.10 ± 46.62 pg/mL and PD: 1435.95 ± 43. 97 pg/mL, *p* < 0.0001) and 28 (NPD:1237.93 ± 25.18 pg/mL and PD: 1382.95 ± 36.73 pg/mL, *p* = 0.0170). No significant difference between groups was observed at week 32 (*p* = 0.9010). However, the dopamine concentration in the PD group (1337.87 ± 32.06 pg/mL) was higher than the concentration in the NPD group (1300.66 ± 30.94 pg/mL).

**Figure 8:**
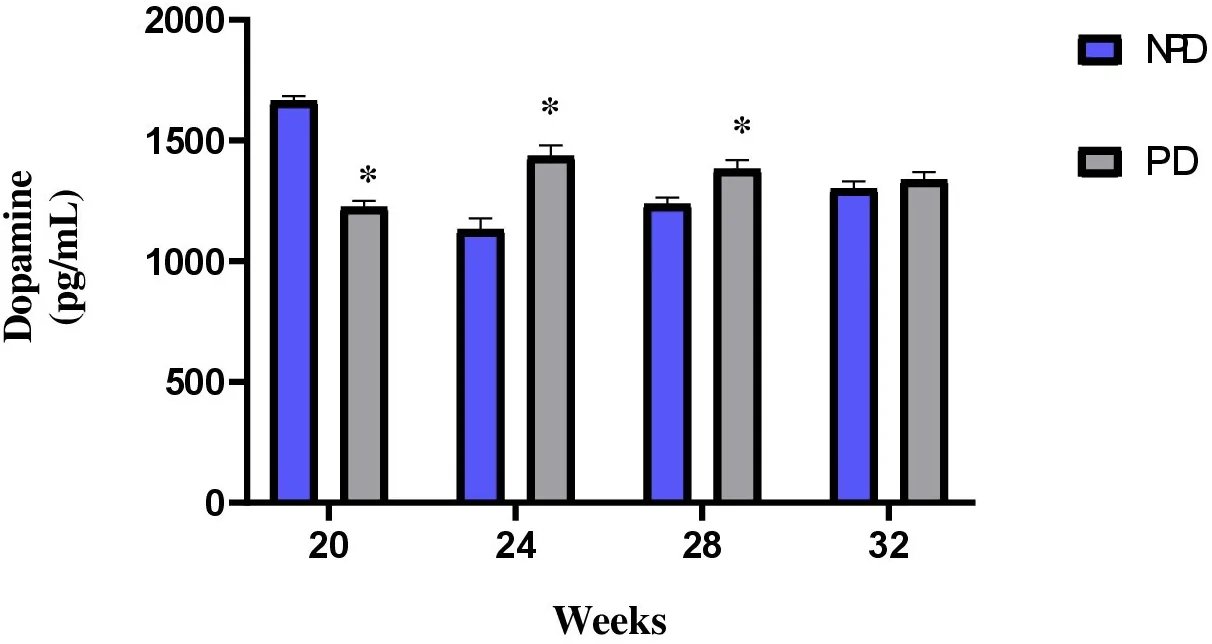
Dopamine concentration in the NPD and PD group (n 6 per group). Values are expressed as mean ± SEM. *Indicates statistical significance. NPD, non-prediabetes; PD, prediabetes.

### Adrenergic receptor (AR)

#### Beta-1-Adrenergic receptor (β_1_-AR) and Beta-2-Adrenergic receptor (β_2_-AR)

A two-way ANOVA was conducted to evaluate the effects of dietary group (NPD vs PD) and time (weeks 20-32) on β1-AR and β2-AR mRNA expression. In **Figure 9A**, a significant interaction between group and time was observed (F (3,40) =25.98, *p* < 0.0001, η²=0.661), indicating that temporal changes in β1-AR expression differed between groups. There was also a significant main effect of time (F (3,40) =299.3, *p* < 0.0001, η²=0.957), while the overall effect of group was not significant (F (1,40) =0.5741, *p* = 0.4531, η²=0.014). Sidak’s *post hoc* comparisons showed no significant difference between groups at week 20 (*p* = 0.2547). At weeks 24 and 28, β_1_-AR expression was significantly downregulated (*p* = 0.0005 and *p* < 0.0001, respectively) in the PD group, whereas it remained unchanged in the NPD group. At week 32, β_1_-AR expression was significantly (*p* < 0.0001) upregulated in the PD group compared with the NPD group. In **Figure 9B**, a significant interaction between group and time was observed (F (3,40) =40.17, *p* < 0.0001, η²=0.751), indicating that temporal changes in β_2_-AR expression differed between groups. Significant main effects of time (F (3,40) =27.04, *p* < 0.0001, η²=0.670) and group (F (1,40) =5.869, *p* = 0.0200, η²=0.128) were also observed. Overall, β_2_-AR mRNA expression was significantly higher in the PD group than in the NPD group (PD: 1.318 and NPD: 1.140; mean difference = 0.179, 95% CI: 0.030-0.328). Sidak’s *post hoc* comparisons revealed that β_2_-AR mRNA expression was significantly higher in the PD group at week 20 (*p* = 0.0004), lower at week 28 (*p* < 0.0001), and higher at week 32 (*p* < 0.0001). No significant difference was observed between the groups at week 24 (*p* = 0.7253).

**Figure 9:**
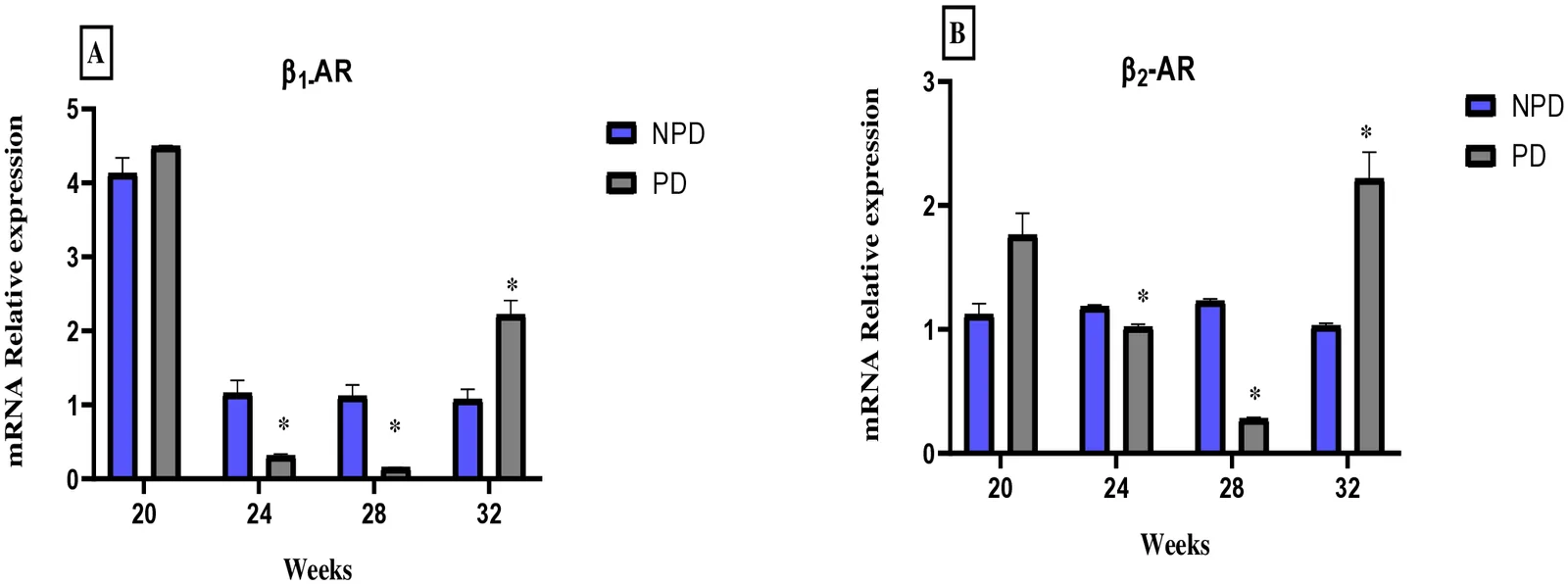
The expression of **(A)** beta-1-adrenergic and **(B)** beta-2-adrenergic receptors in the NPD and PD groups (n = 6 per group). Values are expressed as mean ± SEM. *Indicates statistical significance. NPD, non-prediabetes; PD, prediabetes; B1-AR, beta-1-adrenergic receptor; B2-AR beta-2-adrenergic receptor.

#### Correlation between plasma norepinephrine concentration, hippocampal β1-adrenergic receptor (β1-AR) and β_2_-adrenergic receptor (β_2_-AR) mRNA expression.

**Table 6** shows that at weeks 20 and 24, neither β1-AR nor β2-AR had significant correlations with NE in either group. At week 28, however, NE was strongly and positively correlated with β1-AR expression in the PD groups (r = 0.93, 95% CI: 0.51-0.99, *p* = 0.0064). At week 32, in the NPD group, NE was strongly and negatively correlated with β1-AR expression (r = −0.88, 95% CI: -0.99 - -0.25, *p* = 0.0198). After FDR correction, none of the significant correlations remained significant. The β2-AR correlations were not statistically significant at any time point in either group.

**Table 6:** Correlation between plasma NE concentration and hippocampal β1-AR and β_2_-AR expression in the NPD and PD group at week 20, 24, 28 and 32.

Week	Group (n = 6 per group)	Correlation coefficient	95% CI	*p*-value	*q-*value
β1-AR					
20	NPD	ρ = 0.02		*p >*0.999	1.0000
	PD	r = 0.76	-0.12 - 0.97	0.0769	0.3602
24	NPD	r = -0.20	-0.87 - 0.73	0.7094	1.0000
	PD	r = -0.16	-0.86 - 0.75	0.7578	1.0000
28	NPD	r = 0.33	-0.66 - 0.90	0.5208	0.9722
	PD	r = 0.93	0.51 - 0.99	0.0064	0.1075
32	NPD	r = -0.88	-0.99 - -0.25	0.0198	0.2218
	PD	r = 0.40	-0.61 – 0.92	0.4261	0.8948
β_2_-AR					
20	NPD	r = -0.03	-0.83 - 0.80	0.9499	1.0000
	PD	ρ = 0.09		0.9194	1.0000
24	NPD	r = 0.23	-0.72 - 0.88	0.6610	1.0000
	PD	r = -0.13	-0.85 - 0.76	0.8105	1.0000
28	NPD	r = 0.41	-0.60 - 0.92	0.4138	0.8948
	PD	r = 0.76	-0.13 - 0.97	0.0782	0.3602
32	NPD	r = 0.03	-0.80 - 0.82	0.9583	1.0000
	PD	ρ = 0.37		0.4972	0.9722

Data are presented as correlation coefficient, 95% CI, *p*-value and FDR-adjusted *q*-value (n = 6 per group).

#### Correlation between plasma epinephrine concentration, hippocampal β1-adrenergic receptor (β1-AR) and β_2_-adrenergic receptor (β_2_-AR) mRNA expression.

**Table 7** shows that there were no significant associations between EPI and β1-AR expression in either group at weeks 20, 24 and 28. At week 32, in the PD group, EPI showed a very strong positive correlation with β1-AR expression (r = 0.95, 95% CI: 0.60 - 0.99, *p* = 0.0037) but was not significant after FDR correction (*q* = 0.1075). Similarly, the strong positive correlation (r = 0.82, 95% CI: 0.04 – 0.98, *p* = 0.0436) between EPI and β2-AR expression observed at week 20 in the NPD group did not remain significant after FDR correction (*q* = 0.3602).

**Table 7:** Correlation between plasma EPI concentration and hippocampal β1-AR and β_2_-AR expression in the NPD and PD group at week 20, 24, 28 and 32.

Week	Group (n = 6 per group)	Correlation coefficient	95% CI	*p*-value	*q*-value
β1-AR					
20	NPD	ρ = -0.31		0.5639	0.9972
	PD	r = -0.72	-0.97 - 0.21	0.1034	0.3602
24	NPD	r = -0.74	-0.97 - 0.18	0.0939	0.3602
	PD	r = 0.15	-0.76 - 0.86	0.7824	1.0000
28	NPD	r = 0.44	-0.58 - 0.92	0.3834	0.8948
	PD	r = 0.20	-0.73 - 0.87	0.7104	1.0000
32	NPD	r = -0.72	-0.97 - 0.22	0.1072	0.3602
	PD	r = 0.95	0.60 - 0.99	0.0037	0.1075
β_2_-AR					
20	NPD	r = 0.82	0.04 – 0.98	0.0436	0.3602
	PD	ρ = -0.49		0.3556	0.8948
24	NPD	r = -0.56	-0.94 - 0.46	0.2429	0.7006
	PD	r = -0.12	-0.85 - 0.77	0.8250	1.0000
28	NPD	r = 0.05	-0.79 - 0.83	0.9260	1.0000
	PD	r = 0.56	-0.46 - 0.94	0.2502	0.7006
32	NPD	r = -0.26	-0.88 - 0.70	0.6189	1.0000
	PD	ρ = 0.77		0.1028	0.3602

Data are presented as correlation coefficient, 95% CI, *p*-value and FDR-adjusted *q*-value (n = 6 per group).

### Endogenous opioids

#### Beta endorphin

A two-way ANOVA was conducted to evaluate the effects of dietary group (NPD vs PD) and time (weeks 20-32) on β-endorphin concentrations. There was a significant interaction between group and time (F (3,40) = 23.08, *p* < 0.0001, η²=0.634), indicating that temporal changes in β-endorphin concentrations differed between groups. A significant main effect of time was also observed (F(3,40) = 8.933, *p* = 0.0001, η²=0.401), whereas the overall effect of group was not significant (F(1,40) = 0.0001, *p* = 0.9918, η² <0.001). In **Figure 10**, Sidak’s *post hoc* comparisons showed significantly higher β-endorphin concentrations in the NPD group at week 20 (NPD:529.35 ± 9.70 pg/mL and PD:376.72 ± 18.04 pg/mL, *p* < 0.0001), while the PD group exhibited significantly higher concentrations at week 24 (NPD: 409.60 ± 19.27 pg/mL and PD:484.71± 8.2 pg/mL, *p* = 0.0047). No significant differences were observed in weeks 28 or 32 (*p*>0.05).

**Figure 10:**
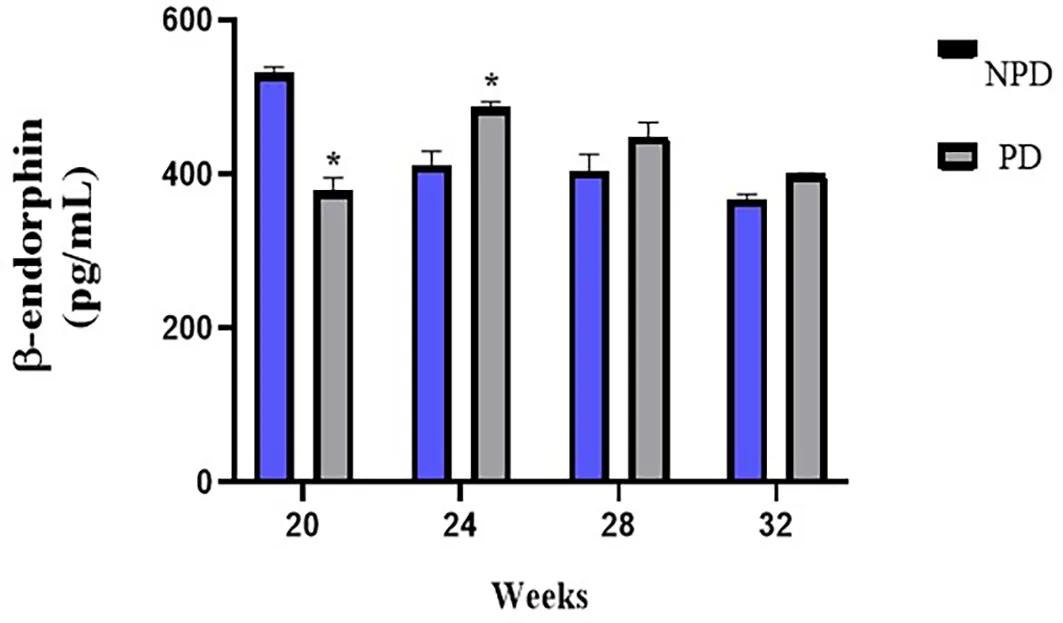
Beta endorphin concentration in the NPD and PD groups (n = 6 per group). Values are expressed as mean ± SEM. *Indicate statistical significance; NPD, non-prediabetes; PD, prediabetes.

### Opioid receptors

#### Mu-Opioid receptor (MOR)

A two-way ANOVA was conducted to determine the effects of dietary group (NPD vs PD) and time (weeks 20-32) on MOR mRNA expression. There was a significant interaction between group and time (F (3,40) =148.3, *p* < 0.0001, η²=0.918), indicating that temporal changes in MOR expression differed between groups. Significant main effects of time (F (3,40) =157.2, *p* < 0.0001, η²=0.922) and group (F (1,40) =312.7, *p* < 0.0001, η²=0.887) were also observed. Overall, MOR expression was significantly higher in the PD group than in the NPD group (PD: 2.798 and NPD: 1.135; mean difference = 1.663, 95% CI: 1.473-1.853). In **Figure 11**, Sidak’s *post hoc* comparisons revealed significantly higher MOR expression in the PD group than in the NPD group at week 20 (*p* < 0.0001), week 24 (*p* = 0.0068), and week 32 (*p* < 0.0001), whereas no significant difference was observed at week 28 (*p* = 0.9998).

**Figure 11:**
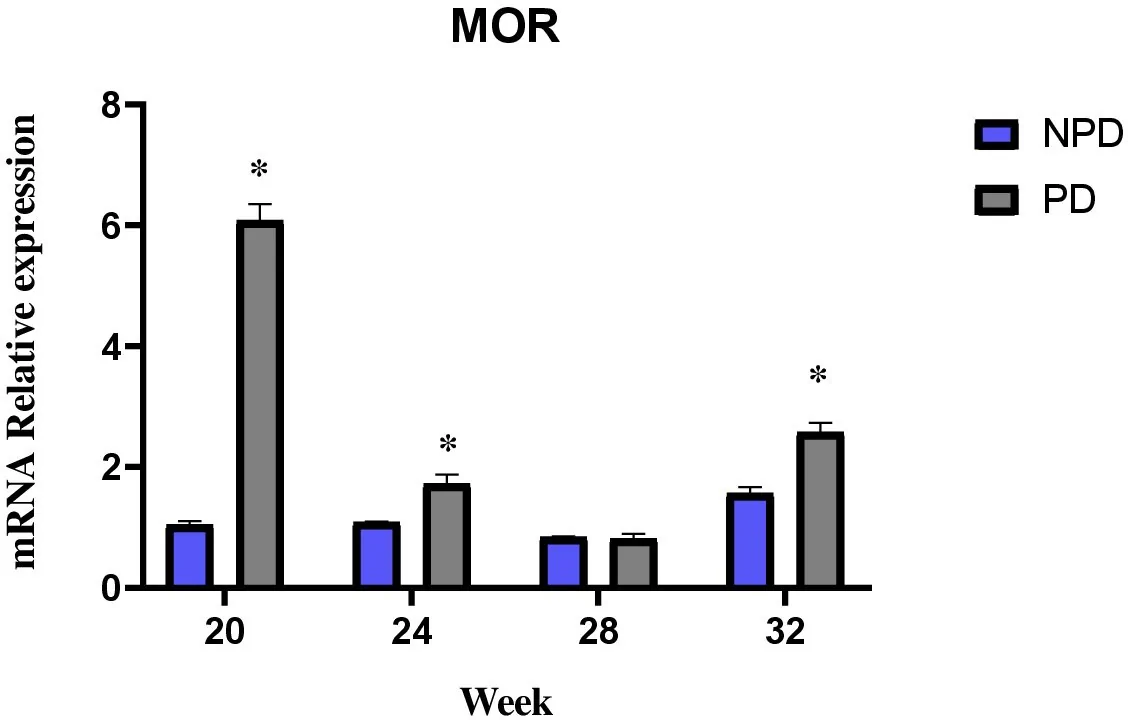
The expression of mu-opioid receptor between the NPD and PD groups (n = 6 per group). Values are expressed as mean + SEM. *Indicates statistical significance. NPD, non- prediabetes; PD, prediabetes; MOR, mu-opioid receptor.

### Delta opioid receptor (DOR) and Kappa opioid receptor (KOR)

A two-way ANOVA was conducted to determine the effects of dietary group (NPD vs PD) and time (weeks 20-32) on DOR and KOR mRNA expression. In **Figure 12A**, there was a significant interaction between group and time (F (3,40) =43.89, *p* < 0.0001, η²=0.767), indicating that temporal changes in DOR mRNA expression differed between groups. Significant main effects of time (F (3,40) =100.2, *p* < 0.0001, η²=0.883) and group (F (1,40) =15.54, *p* = 0.0003, η²=0.280) were also observed. Overall, DOR mRNA expression was significantly higher in the PD group than in the NPD group (PD: 1.869 and NPD: 1.403; mean difference = 0.466, 95% CI: 0.227-0.705). Sidak’s *post hoc* comparisons revealed significantly higher DOR expression in the PD group at weeks 20 and 32 (both p<0.0001), whereas significantly lower mRNA expression was observed compared with the NPD group at weeks 24 (*p* = 0.0023) and 28 (*p* = 0.0025). In **Figure 12B**, there was a significant interaction between group and time (F (3,40) =158.8, *p* < 0.0001, η²=0.923), indicating that temporal changes in KOR expression differed between groups. Significant main effects of time (F (3,40) =155.5, *p* < 0.0001, η²=0.921) and group (F (1,40) =243.0, *p* < 0.0001, η²=0.859) were also observed. Overall, KOR mRNA expression was significantly higher in the PD group than in the NPD group (PD: 2.430 and NPD: 1.101; mean difference = 1.329, 95% CI: 1.157-1.501). Sidak’s *post hoc* comparisons revealed significantly higher KOR mRNA expression in the PD group at weeks 20 and 32 (both *p* < 0.0001), whereas the NPD group exhibited significantly higher KOR mRNA expression at week 28 (*p* = 0.0005). No significant difference was observed at week 24 (*p* = 0.9762).

**Figure 12:**
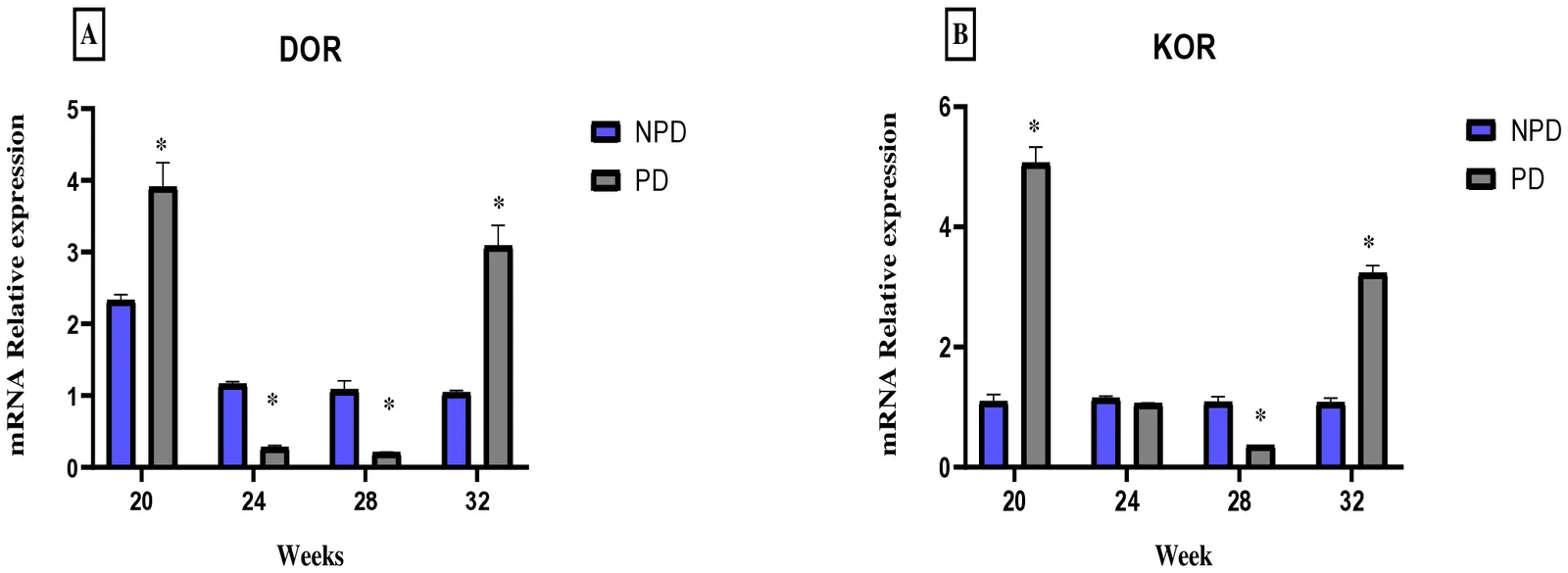
The expression of **(A)** delta-opioid and **(B)** kappa-opioid receptors between the NPD and PD group. Values are expressed as mean ± SEM. *Indicates statistical significance. NPD, non- prediabetes; PD, prediabetes; DOR, delta-opioid receptor; KOR, kappa-opioid receptor.

#### Correlation between plasma β-endorphin concentration, hippocampal µ-opioid (MOR), δ-opioid (DOR) and κ-opioid receptor (KOR) mRNA expression.

**Table 8** shows that there were no significant correlations between β-endorphin and MOR and with DOR at any time point in either group. At week 32, in the NPD group, β-endorphin and KOR showed an almost perfect negative correlation (r = −1.00, 95% CI: -0.99 - -0.98, *p* < 0.0001). In contrast, the PD group demonstrated a strong positive correlation (r = 0.86, 95% CI: 0.15-0.98, *p* = 0.0292). Only the NPD group β-endorphin to KOR correlation remained significant after FDR correction (*q* = 0.0024).

**Table 8:** Correlation between the plasma beta-endorphin concentration and hippocampal MOR, KOR and DOR mRNA expression in the NPD and PD group at week 20, 24, 28 and 32.

Week	Group (n = 6 per group)	Correlation coefficient	95% CI	*p*-value	*q*-value
MOR					
20	NPD	r = 0.46	-0.56 - 0.93	0.3625	0.7295
	PD	r = -0.38	-0.91 - 0.63	0.4593	0.7775
24	NPD	r = 0.07	-0.78 - 0.84	0.8880	0.8991
	PD	ρ = -0.37		0.4972	0.7775
28	NPD	r = 0.4	-0.60 - 0.92	0.4137	0.7685
	PD	r = 0.15	-0.75 - 0.86	0.7702	0.8991
32	NPD	r = -0.12	-0.85 - 0.77	0.8166	0.8991
	PD	r = 0.25	-0.70 - 0.88	0.6302	0.8455
DOR					
20	NPD	r = -0.48	-0.93 - 0.54	0.3328	0.7295
	PD	r = 0.75	-0.17 - 0.97	0.0888	0.4289
24	NPD	r = 0.77	-0.12 - 0.97	0.0745	0.4289
	PD	r = -0.62	-0.95 - 0.39	0.1925	0.6222
28	NPD	r = 0.10	-0.77 - 0.84	0.8485	0.8991
	PD	r = 0.34	-0.65 - 0.90	0.5151	0.7775
32	NPD	r = -0.53	-0.94 - 0.49	0.2795	0.7295
	PD	r = 0.46	-0.56 - 0.92	0.3617	0.7295
KOR					
20	NPD	r = 0.79	-0.06 - 0.98	0.0605	0.4289
	PD	r = 0.23	-0.72 - 0.88	0.6663	0.8469
24	NPD	r = 0.71	-0.23 - 0.97	0.1105	0.4448
	PD	r = 0.60	-0.41 - 0.95	0.2061	0.6222
28	NPD	r = 0.07	-0.79 - 0.83	0.8935	0.8991
	PD	r = -0.29	-0.89 - 0.68	0.5789	0.8224
32	NPD	r = -1	-0.99 - -0.98	<0.0001	0.0024
	PD	r = 0.86	0.15 - 0.98	0.0292	0.3526

Data are presented as correlation coefficient, 95% CI, *p*-value and FDR-adjusted *q*-value (n = 6 per group).

## Discussion

The hypothalamus-pituitary-adrenal (HPA) axis and the sympathetic nervous system (SNS) are neuroendocrine systems that maintain homeostasis during physiological and metabolic challenges (24). The endogenous opioid system has a role in the regulation of stress responses, with β-endorphin exerting inhibitory effects on corticotropin-releasing hormone (CRH) and the central locus coeruleus/norepinephrine (LC/NE) system, thereby regulating the function of the HPA-axis and SNS activity (10). In T2DM, chronic hyperglycemia is associated with the hyperactivation and dysregulation of both the HPA-axis and SNS (25). Prediabetes precedes T2DM; characterizing neuroendocrine alterations during this period may provide insight into the physiological adaptations accompanying metabolic deterioration (26). It has been established that the HPA-axis is dysregulated during prediabetes and there is a high prevalence of SNS dysfunction among individuals with impaired fasting glucose (IFG) (21, 22). Although HPA-axis and SNS alterations have been described during prediabetes, the endogenous opioid system has received comparatively less attention, and few studies have examined these systems collectively during the progression of prediabetes. Therefore, the present study investigated temporal changes in the HPA axis, SNS, and endogenous opioid system in male Sprague-Dawley rats with HFHC diet-induced prediabetes.

The consumption of an HFHC diet has been shown to induce metabolic characteristics of prediabetes in Sprague-Dawley rats, thus creating an animal model that mimics the human condition of prediabetes (27). Animals on an HFHC diet show early defects in insulin secretion and insulin peripheral sensitivity, leading to elevated blood glucose levels (28). In previous studies, animals on an HFHC diet developed prediabetes characteristics such as increased body weight gain, FBG, impaired glucose tolerance and elevated HbA_1c_ (27, 29, 30). Consistent with previous studies, HFHC-fed animals exhibited increased body weight and FBG, impaired glucose tolerance, and higher HbA1c compared with the animals on a standard diet. Importantly, HbA1c demonstrated a strong main effect of dietary group, with consistently higher concentrations in the PD group from week 20 through week 32, despite the absence of a significant group × time interaction. This suggests that prediabetes was successfully induced and the condition remained throughout the weeks.

### HPA-axis changes in prediabetes

The HPA-axis system is essential for stress adaptation and promotes the mobilization of energy through glucocorticoid secretion (1). The HPA-axis is regulated by an integrated network involving excitatory and inhibitory inputs to the paraventricular nucleus (PVN) CRH neurons, including brainstem, hypothalamic, amygdala, hippocampal, and prefrontal cortical pathways (31, 32). Consequently, alterations in circulating glucocorticoids may reflect not only changes in hypothalamic or pituitary drive but also altered feedback regulation within the broader neural circuitry controlling the HPA axis. In the present study, at week 20, both ACTH and CORT concentrations were significantly lower in the PD group than in the NPD group. This finding differs from previous work in which diet-induced prediabetic rats exhibited increased ACTH and CORT at week 20, followed by lower ACTH and higher CORT at week 32 (21). The differences may reflect differences in sampling or the temporal dynamics of HPA-axis adaptation. Importantly, the present study demonstrated significant group × time interactions for both ACTH and CORT, indicating that HPA-axis activity did not differ between the metabolic groups in a uniform manner but changed differently over time.

CORT concentrations subsequently became higher in the PD group at weeks 24 and 32, despite the absence of significant between-group differences in ACTH during these weeks. This dissociation between ACTH and CORT may indicate that adrenal glucocorticoid output becomes less tightly coupled to circulating ACTH as metabolic dysfunction persists. However, because adrenal sensitivity to ACTH, CRH secretion, and HPA-axis feedback were not directly assessed, altered pituitary to adrenal coupling cannot be established from the present findings. Rather, the results suggest that sustained metabolic stress is associated with temporal disruption of the normal coordination between ACTH and CORT secretion.

This interpretation is supported by correlation analysis. A strong positive ACTH-to-CORT association was observed in the NPD group at week 20, consistent with coordinated pituitary stimulation of adrenal glucocorticoid secretion; however, this association did not remain significant following FDR correction. No significant ACTH-to-CORT associations were detected in the PD group. Correlation findings should be interpreted with caution due to multiple comparisons and a limited sample size.

### Changes in hippocampal GR and MR expression.

Glucocorticoids exert their function by binding to the glucocorticoid receptor (GR) and the mineralocorticoid receptor (MR) (33). MRs have a high affinity for corticosteroids; they remain substantially occupied under basal conditions and are primarily responsible for setting the threshold for stress responsiveness (34). GRs, by contrast, possess lower binding affinity and are heavily recruited during peaks in plasma corticosteroid concentrations, typically induced by acute stressors, thereby playing an essential role in stress recovery (35). The hippocampus is important for glucocorticoid feedback regulation and is sensitive to metabolic disturbances (36). Hyperglycemic conditions, such as T2DM, cause localized overexpression and hyperactivation of the GRs in the hippocampal CA1 region (36, 37). Previous studies have reported a significant reduction in central MR and GR expression following chronic high-fat feeding, suggesting that prolonged dietary stress can alter glucocorticoid receptor regulation (38, 39). In contrast, the present findings demonstrate a distinctly time-dependent trend. GR expression was increased in the PD group at week 20, whereas both GR and MR mRNA expression were reduced at weeks 24 and 28, then increased at week 32. The reduction in hippocampal GR and MR mRNA observed in the present study may represent an adaptive transcriptional response to prolonged metabolic and glucocorticoid exposure. Chronic glucocorticoid exposure can alter glucocorticoid receptor signaling and contribute to changes in metabolic and cellular responsiveness (40, 41). However, a direct causal relationship between circulating CORT and receptor downregulation cannot be established from the present data. The present findings, therefore, indicate temporal changes in receptor gene expression rather than definitive alterations in receptor sensitivity or feedback function.

By week 32, both hippocampal GR and MR mRNA expression levels were significantly higher in the PD group than in the NPD group. Our findings are in contrast with those of Mosili et al., who reported downregulation of both GR and MR in the PD group (21). This difference further supports the possibility that glucocorticoid receptor regulation during prediabetes is dynamic rather than unidirectional. In the present study, correlation analysis revealed that both GR and MR expression were strongly negatively associated with circulating CORT in the PD group at week 32. The associations did not remain significant after FDR correction. These findings suggest that the increased GR and MR expression transcription is influenced by factors beyond circulating CORT concentration. The functional significance of this increase remains uncertain because receptor protein levels and receptor activity were not determined. Furthermore, the present study focused on hippocampal GR and MR expression, whereas HPA-axis regulation involves several brain regions, including the hippocampus, prefrontal cortex, and amygdala, and receptor responses may differ by brain region and by the duration of metabolic or psychological stress (42). Overall, the present findings suggest that hippocampal GR and MR transcription undergoes dynamic changes during sustained prediabetes, supporting a model of stage-dependent adaptation rather than a simple progressive increase or decrease in glucocorticoid signaling.

### Changes in plasma catecholamine concentration and hippocampal adrenergic receptors during prediabetes.

The activation of the HPA axis occurs concurrently with the activation of the sympathetic nervous system (SNS), leading to the simultaneous release of glucocorticoids and catecholamines, including norepinephrine (NE) and epinephrine (EPI) (43). NE elevates blood glucose by enhancing gluconeogenesis, whereas EPI drives this elevation by promoting glycogenolysis (44). Dopamine also participates in metabolic regulation, including effects on insulin secretion and glucose homeostasis (45). In overt T2DM, chronic hyperglycemia stimulates excessive catecholamine secretion (46). Chronic overstimulation of the SNS and the resulting high catecholamine concentrations have also been implicated in the development of metabolic and cardiovascular complications associated with diabetes (47). A study evaluating plasma concentrations of NE, EPI, and dopamine found higher levels in patients with newly diagnosed T2DM than in those with prediabetes (48). Conversely, another clinical evaluation reported that free urinary EPI is elevated in obese individuals and at the onset of diabetes, while concurrent levels of NE and dopamine are decreased (49). Mosili et al. reported no significant difference in NE and EPI concentrations between NPD and PD patients (54). In the present study, the PD group exhibited marked temporal changes in NE, EPI and dopamine concentrations. NE concentration was lower in the PD group at weeks 20 and 28 but became significantly higher at week 32. Plasma EPI concentration was unchanged at weeks 20 and 24, lower in PD groups at week 28, and markedly higher at week 32. Dopamine concentration showed an early reduction in the PD group at week 20, followed by elevations at weeks 24 and 28 and convergence between groups at week 32. The significant group × time interactions observed for these catecholamines suggest that prediabetes was associated with a temporal reorganization of catecholaminergic activity rather than a consistent increase in sympathetic hormone concentrations.

The lower NE concentration observed early in the prediabetic period is consistent with evidence that autonomic responses to metabolic disturbances may differ between prediabetes and established T2DM (50). Conversely, the marked elevation of NE and EPI at week 32 suggests that prolonged metabolic dysfunction may eventually be accompanied by increased sympathetic activation. This later catecholaminergic elevation is also consistent with evidence linking chronic sympathetic activation with metabolic and cardiovascular complications in diabetes (51). However, circulating catecholamine concentrations provide an indirect measure of sympathetic activity and may be influenced by secretion, metabolism, clearance, and tissue uptake. Therefore, the present findings demonstrate altered systemic catecholamine availability rather than directly proving generalized sympathetic overactivity.

The temporal pattern observed for dopamine further emphasizes that individual catecholamines may not respond uniformly to metabolic stress. The early reduction, followed by a transient elevation, of dopamine may reflect stage-dependent changes in dopaminergic regulation of metabolic homeostasis. Previous clinical studies have similarly reported alterations in catecholamine regulation across prediabetic and diabetic states (48–50). This inconsistency may reflect differences in disease stage, metabolic phenotype, sampling conditions, and the physiological context in which catecholamines were assessed.

To determine whether the temporal changes in circulating catecholamines were accompanied by alterations in downstream adrenergic signaling capacity, hippocampal β1-AR and β2-AR mRNA expression was assessed. β_1_-AR and β_2_-AR mediate distinct intracellular signaling pathways (52). In the hippocampus, β-adrenergic signaling contributes to synaptic plasticity and memory-related processes, whereas peripheral β-adrenergic signaling influences metabolic function (53, 54). In the present study, β1-AR expression was reduced in the PD group at weeks 24 and 28 but became markedly elevated at week 32, whereas β2-AR expression showed a fluctuating pattern, with higher expression at weeks 20 and 32 and lower expression at week 28. These findings suggest that adrenergic receptor regulation was highly dynamic during the progression of prediabetes. The concurrent elevation of catecholamines and adrenergic receptor expression suggests that sympathetic signaling may be altered at this later stage of prediabetes (55). Experimental diabetes has previously been associated with alterations in brain β-adrenergic receptor density and NE metabolism (56). These findings support the possibility that metabolic disturbances may produce compensatory or stage-dependent changes in hippocampal adrenergic receptor regulation.

NE was strongly positively associated with β1-AR expression in the PD group at week 28, while EPI showed a very strong positive association with β1-AR expression in PD animals at week 32. However, neither association remained significant following FDR correction. The observed correlations indicate that circulating catecholamine concentrations and hippocampal adrenergic receptor transcript abundance covaried at selected time points. However, these associations do not establish altered sympathetic signaling or receptor function.

### Changes in endogenous opioid hormones and receptors during prediabetes.

The endogenous opioid system represents an additional component of the neuroendocrine response to metabolic and physiological stress. β-endorphin is derived from proopiomelanocortin (POMC), the same precursor from which ACTH is produced, and can regulate both the HPA-axis and sympathetic activity (10, 57). β-endorphin acts predominantly through the μ-opioid receptor (MOR), whereas endogenous opioid signaling more broadly involves the MOR, κ-opioid receptor (KOR), and δ-opioid receptor (DOR) pathways (58). In addition to neuroendocrine effects, β-endorphin plays a role in glucose regulation through effects on insulin signaling and GLUT4-mediated glucose uptake (18, 19).

Elevated circulating β-endorphin levels have previously been associated with alterations in glucose regulation, while clinical studies have reported reductions in systemic β-endorphin and peripheral MOR expression in overt T2DM (17, 59–61). Conversely, within the prediabetic state, Moustafa and colleagues reported that β-endorphin and endomorphin-2 (EM2), MOR and KOR are elevated in patients with prediabetes compared to normoglycemic patients (62). In that study, β-endorphin, MOR and KOR were particularly associated with insulin resistance and inflammatory status, providing evidence that endogenous opioid signaling is altered before the development of overt T2DM (62). These findings suggest that alterations in opioid signaling within the hippocampus may occur before the development of overt diabetes.

In the present study, β-endorphin concentrations were lower in the PD group at week 20 but increased at week 24. The transient increase coincided with changes in glucose and other stress-related mediators. In hyperglycemic conditions, elevated β-endorphin levels have been proposed to influence glucose regulation (63). In addition, hippocampal MOR, DOR and KOR expression demonstrated marked group × time interactions, with particularly large effect sizes for MOR and KOR, suggesting substantial alteration of hippocampal opioid receptor expression in response to metabolic dysfunction.

Although few studies have examined all three opioid receptors at different time points during prediabetes, these findings are broadly consistent with those of Moustafa and colleagues, who reported increased β-endorphin, MOR and KOR in individuals with prediabetes and demonstrated associations between endogenous opioid markers, insulin resistance and inflammatory mediators (62). The concurrent reduction in circulating β-endorphin and increase in receptor mRNA expression observed in the present study may represent a compensatory alteration intended to maintain opioid signaling despite reduced ligand availability. However, this interpretation remains speculative because receptor protein abundance, receptor occupancy, and downstream signaling were not assessed. Moreover, increased receptor mRNA does not necessarily indicate increased receptor density or enhanced receptor responsiveness.

By week 32, both β-endorphin concentration and hippocampal opioid receptor mRNA expression were elevated in the PD group. In the NPD group, β-endorphin was very strongly and inversely correlated with KOR expression, and this association remained significant after FDR correction. This inverse relationship is compatible with a potential ligand–receptor homeostatic mechanism, in which increased endogenous ligand availability is associated with reduced receptor expression (64, 65). Interestingly, the direction of the association was reversed in the PD group, where β-endorphin and KOR expression showed a strong positive correlation; however, this association did not survive FDR correction. Therefore, the apparent divergence between the NPD and PD groups should not be interpreted as definitive evidence of altered β-endorphin-to-KOR coupling. Rather, it represents an exploratory observation that may indicate altered regulation of the endogenous opioid system during metabolic dysfunction. However, the results should be interpreted with caution due the small number of animals per group therefore, further studies with larger sample size are warranted to confirm the present findings. Opioid receptors are subject to multiple regulatory processes, including phosphorylation, β-arrestin recruitment, internalization, and changes in receptor availability (55, 65, 66). The simultaneous increase in β-endorphin and hippocampal opioid receptor transcripts at week 32 may therefore reflect a broader adaptive response of the endogenous opioid system to prolonged metabolic stress.

## Conclusion

The present study demonstrates that HFHC diet-induced prediabetes is associated with progressive changes in the HPA axis, SNS, and endogenous opioid system that become more pronounced with disease progression. Changes in circulating ACTH, CORT, NE, EPI, dopamine, and β-endorphin were accompanied by temporal alterations in the expression of their corresponding receptors, including GR, MR, β1-AR, β2-AR, MOR, DOR, and KOR as measured specifically in the hippocampus. The findings suggest that neuroendocrine hormone and hippocampal receptor expression do not respond uniformly throughout prediabetes but undergo stage-dependent alterations that may reflect adaptation to sustained metabolic stress. Early prediabetes was characterized by reduced ACTH, CORT, NE, and β-endorphin, together with alterations in hippocampal glucocorticoid and adrenergic receptor expression. During the intermediate period, hippocampal receptor expression and circulating catecholamine concentrations fluctuated considerably. By week 32, several neuroendocrine mediators, including CORT, NE, EPI, and β-endorphin, were elevated in PD animals, accompanied by increased hippocampal GR, MR, β1-AR, β2-AR, MOR, DOR, and KOR. This pattern suggests that sustained metabolic dysfunction may progressively alter the relationship between circulating neuroendocrine mediators and receptor expression within the hippocampus. Importantly, these findings should not be interpreted as evidence that similar receptor changes occur throughout the brain. The present study measured receptor mRNA expression in the hippocampus only; therefore, the observed changes cannot be extrapolated to other brain regions involved in HPA-axis, SNS, or endogenous opioid regulation.

Furthermore, the present study cannot determine whether the observed changes represent compensatory adaptations, pathological dysregulation, or a combination of both. The correlation analyses provide evidence of covariation between selected circulating mediators and receptor transcripts, but do not establish directionality or causality. The loss of several associations following FDR correction further emphasizes the need for cautious interpretation. Nevertheless, the consistent temporal changes across these interconnected systems suggest that prolonged prediabetic metabolic stress is accompanied by dynamic changes in the circulating glucocorticoid hormones, catecholamines, β-endorphin, and hippocampal receptor expression.

### Limitations and future studies

A limitation of the present study is the relatively small number of animals per group (*n* = 6). Larger studies are warranted to confirm the present findings and determine whether non-significant outcomes reflect the absence of a meaningful biological effect or limited statistical power. Only receptor mRNA expression was measured. Protein expression was not assessed to see receptor protein abundance and functionality. Consequently, the observed temporal associations between circulating hormones and receptor expression should be interpreted as changes in neuroendocrine regulation rather than direct evidence of altered receptor sensitivity or impaired negative feedback. Receptor expressions were assessed only in the hippocampus. Consequently, the findings cannot be generalized to receptor regulation throughout the central neuroendocrine network. Future studies should assess additional relevant regions, including the hypothalamus, amygdala, prefrontal cortex and brainstem nuclei, to determine whether the observed hippocampal transcriptional changes occur within a broader neural response to metabolic dysfunction. Catecholamine measurements should be interpreted within the limitations of the assay methodology, and confirmation using chromatographic methods such as HPLC or LC-MS/MS would provide additional analytical validation. The present study examined neuroendocrine alterations during diet-induced prediabetes in male Sprague-Dawley rats. Therefore, the findings should not be directly generalized to females or to human prediabetes without further investigation. Additionally, while we have reported on the HPA-axis, SNS, and the endogenous opioid system changes that occur in diet-induced prediabetes, it is crucial that future studies investigate how these systems respond in the presence of diet-induced prediabetes and stress.

## Data Availability

The data generated and analyzed in the current study are not publicly available because additional analyses from the study are ongoing. However, the datasets are available from the corresponding author upon reasonable request.
