# Supplementary material for: Investigating the effect of diet-induced prediabetes on the HPA-axis, sympathetic nervous system and endogenous opioids in male Sprague-Dawley rats
Source: Front Endocrinol (Lausanne). 2026 Sep 29;17:1925577. doi: 10.3389/fendo.2026.1925577 (PMC13657940; doi:10.3389/fendo.2026.1925577)
Supplement: Supplementary file 1 [file SupplementaryFile1.docx]

# 1 Summary of the Results

**Figure 1** below shows the overall results of the study.

**Figure 1**: Summary of the results obtained in the NPD and PD group from week 20, 24, 28 and 32.
